# Efficacy of abdomen-rubbing qigong exercise for chronic insomnia: study protocol for a randomized controlled trial

**DOI:** 10.1186/s13063-021-05528-7

**Published:** 2021-11-06

**Authors:** Chong Guan, Ziji Cheng, Fangfang Xie, Ruiping Wang, Jing Zhang, Fei Yao, Min Fang

**Affiliations:** 1 Shanghai University of Traditional Chinese Medicine, Shanghai, 201203 China; 2 Shanghai Skin Disease Hospital, Shanghai, 200050 China; 3 Institute of Tuina, Shanghai Institute of Traditional Chinese Medicine, Shanghai, 200437 China

**Keywords:** Chronic insomnia, Traditional Chinese medicine, Abdomen-rubbing qigong exercise, Functional magnetic resonance imaging, Randomized controlled trials

## Abstract

**Background:**

Insomnia is a common sleeping disorder which affects the quality of life which can bring harms to physical and mental health of human beings and even economic development. Traditional Chinese medicine (TCM) plays an outstanding role in treating chronic diseases and alleviating their symptoms. Therefore, the purpose of this study is to assess the treatment efficacy in patients with insomnia treated with abdomen-rubbing qigong exercise (ARQE). In addition, the brain function changes of patients will be explored by resting state functional magnetic resonance imaging (rs-fMRI).

**Method/design:**

This trial is a randomized, single-blind, controlled study planned to transpire between July 1, 2020, and July 31, 2021. A sample size of 114 participants (57 per group) with chronic insomnia will be randomly assigned to receive ARQE or CBTI for 8 weeks. The study duration will be 13 weeks, including a 1-week screening period, 8 weeks of intervention, and another 4 weeks of follow-up. The primary outcome is the Pittsburgh sleep quality index scores. Secondary outcomes include insomnia severity index, gastrointestinal symptom rating scale, the Hamilton Depression Scale, and rs-fMRI scan. The adverse events will be in control.

**Discussion:**

The results of this study will help to clarify the efficacy of ARQE in the treatment of insomnia and try to use rs-fMRI technology to explore the brain function changes of ARQE in improving sleep quality in patients with insomnia disorder. If the results are as expected, this study will provide high-quality evidence for the treatment of insomnia with ARQE.

**Trial registration:**

China Clinical Registration Agency ChiCTR1900028009. Registered on 19 December 2019.

## Background

Chronic insomnia refers to a sleeping disorder characterized by frequent or persistent difficulty in falling asleep or staying asleep [1]. Therefore, patients with insomnia hardly feel satisfied with their sleep quality [2]. Insomnia is a worldwide problem. Researches show that the incidence rate in Japan is 37.2%, in the USA is 27.1%, and in China is 15.0% [3, 4]. Insomnia can lead to daytime sleepiness, impaired attention, memory and other cognitive functions, reduced quality of life, and increased risk of medical conditions such as high blood pressure, depression, and anxiety [5–11]. The economic cost of insomnia is enormous, with an estimated of $30 billion to $35 billion spent on insomnia annually in the USA [12]. In conclusion, chronic insomnia is a serious illness which can bring harms to physical and mental health of human beings and even economic development.

Hypnotics and sedatives can relieve the symptoms of insomnia to some extent, but there are risks of adverse reactions and drug addiction. Cognitive behavior therapy of insomnia (CBTI) can change patients’ wrong sleep cognition and behavior, rebuild proper sleep cognition mode, and improve sleep mode [13–15]. It is considered as the cornerstone therapy in insomnia treatment. However, due to the insufficient number of CBTI psychiatrists/psychologists who are well trained in treating insomnia, also only a few patients can afford the treatment bills [16, 17]. Therefore, it is necessary to explore a more economic intervention for patients with insomnia.

The abdomen-rubbing qigong exercise (ARQE) has a long history of treating insomnia in China, which also named “nine rotating methods to prolong life,” summarized by the famous Qing dynasty medical practitioner Fang Kai in China [18]. It was first recorded in the “Nei Gong Tu Shuo” published in 1881 [19]. In China, abdominal massage has been widely used in the treatment of chronic diseases such as digestion diseases and insomnia [20]. Tan’s research shows that abdominal massage can significantly increase the insomnia patient’s total sleep time and lower the PSQI score [21]. ARQE is often used in the clinic of our research group; we found that this intervention is proved to be able to improve not only sleep quality but also gastrointestinal function, which are strongly connected. The beneficial effect of ARQE in chronic insomnia is widely known, but there have been no large-scale randomized controlled trials of efficacy and safety of ARQE treatment and lack of high-quality evidence for the treatment of insomnia with ARQE.

Chronic insomnia is a typical psychosomatic disease, which not only has a negative impact on physical health, but also has a certain impact on the patient’s psychology. Patients with chronic insomnia are often accompanied by emotional disorders such as excessive thinking, anxiety, depression, abnormal feelings, consciousness, emotions, and cognitive functions. Over time, pathological changes in brain structure and compensatory changes will occur [22]. Rs-fMRI (resting-state functional magnetic resonance imaging) study may help in elucidating the brain’s intrinsic functional organization to aid in understanding the brain function changes of insomnia and its treatment. Functional connectivity (FC) and amplitude of low-frequency amplitude (ALFF) are two methods of rs-fMRI analysis. In previous rs-fMRI studies, compared with healthy people, insomnia patients often have abnormalities in part of the brain functional activities and functional connectivity of brain regions. These abnormal areas are mainly concentrated in the prefrontal lobe, hippocampus, temporal cortex, and anterior cingulate [23–25].

Therefore, we aim to conduct a randomized controlled clinical trial of ARQE treatment for chronic insomnia to certify the efficacy of ARQE in the treatment of insomnia and try to use rs-fMRI technology to explore the changes in brain function of ARQE in improving the sleep quality of insomnia patients.

## Method and design

### Study design

**Fig. 1:**
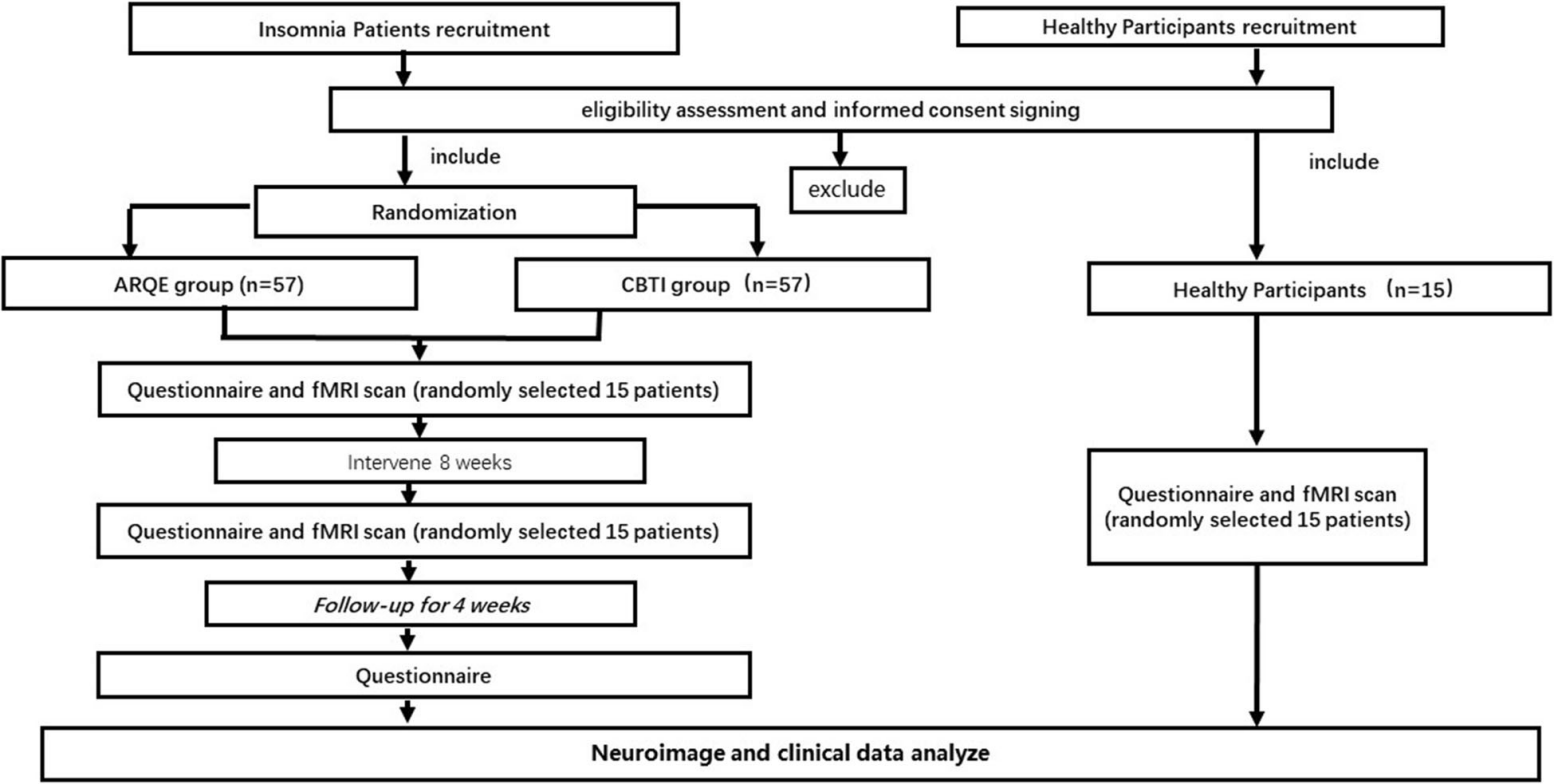
Neuroimage and clinical data analysis

To evaluate the efficacy of ARQE, a clinical randomized controlled trial is designed, with both evaluators and participants blinded to the design. In this trial, the participants are randomly divided into ARQE group and CBTI group, with 57 people in each group. Informed consent will be required for study participation. In addition, all researchers will be required to sign the commitment to protect the confidentiality of study participants. The purpose of the study and possible risks (such as adverse clinical outcomes and treatment-related adverse events) will be informed to every patient. Signed and dated consent forms will be required from those who agree to share their clinical data with the participating hospitals or regulatory agencies. All concomitant care and interventions are permitted other than concomitant receipt of any other experimental treatment. The details are shown in Fig. 1 and Table 1. This SPIRIT checklist is described in detail in additional file 1.

**Table 1:** Schedule for enrollment, intervention, and assessment

Activity	Phase (time point)	Screening/enrolment (week0)	Allocation	Treatment/intervention								Follow-up
				Weeks 1	Weeks 2	Weeks 3	Weeks 4	Weeks 5	Weeks 6	Weeks 7	Weeks 8	Weeks 12
Enrolment	Eligibility screening	√										
	Acquisition of informed consent	√										
	Clinicopathological evaluation	√										
	Enrolment	√										
	Random allocation		√									
Treatment/intervention	Abdomen-rubbing qigong exercise			√	√	√	√	√	√	√	√	
	Cognitive behavioral therapy for insomnia			√	√	√	√	√	√	√	√	
Outcome assessment	PSQI	√					√				√	√
	ISI	√					√				√	√
	GSRS	√					√				√	√
	HAMD	√					√				√	√
Safety observation	fMRI	√									√	
	Adverse event			√	√	√	√	√	√	√	√	√
	drug combination			√	√	√	√	√	√	√	√	√

### Participants

#### Recruitment strategy

Participants will be recruited through advertisement and referrals starting in July 1, 2020. There are three main strategies for recruiting insomniacs. The first strategy was to recruit participants from the outpatient and inpatient departments of Yueyang Hospital of Integrated Traditional Chinese and Western Medicine affiliated to Shanghai University of Traditional Chinese Medicine. Second, printed recruitment posters will be distributed at the Shanghai university of traditional Chinese medicine and in nearby communities to recruit potential research subjects. Third, we will advertise our research through the Internet and social media (e.g., WeChat) to attract patients who may be willing to participate.

#### Sample size calculation

The required sample size is calculated using an estimation formula based on the difference between two sample means and standard deviations (Pittsburgh Sleep Quality Index, which is assessed using composite points of sleep quality). A previous clinical study suggests that the mean and standard deviations of Pittsburgh Sleep Quality Index (PSQI) in ARQE group(*n* = 40) after treatment are 8.45 and 2.06, while the mean and standard deviations of PSQI in CBTI group (*n* = 40) after treatment are 9.92 and 2.65 [26, 27]. The sample size is calculated in the following formula, with the two-sided significance level (α) set at 0.05 and statistical power at 0.80 [28]. Thus, a minimum sample size of 51 participants per group (102 participants in total) is calculated to provide sufficient statistical power to detect a between-group mean difference of 1.47 in treatment efficiency, defined as the change in PSQI score.

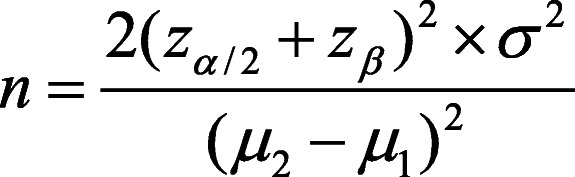



Considering a 10% loss to follow-up, we aim to recruit 57 patients in each group, in a total of 114 patients. According to fMRI studies, 15 to 30 patients are adequate to test hypotheses.

### Inclusion criteria for patients with insomnia

Participants who meet all the following criteria can be enrolled: [1] meeting the diagnostic criteria of ICSD-3 criteria of insomnia [2]; ISI scale score between 8 to 21 [3]; 20 to 45 years old, male or female [4]; HAMD score less than or equal to 17 [5]; not having received any treatments for insomnia for the past month, including medication, and acupuncture [6]; normal result of blood routine [7]; right-handed; and [8] understanding the process of this study and agreeing to participate and sign the consent form.

### Exclusion criteria for patients with insomnia

Participants with any one of the following conditions will be excluded from this trial: [1] experience major depressive disorder, anxiety disorder, panic disorder, or other mental disorders or alcohol or drug addiction [2]; having irregular circadian cycle due to work requirement, [3] insomnia caused by severe pain or other underlying diseases [4]; having problems with communication and unable to perform abdominal rubbing [5]; experience insomnia caused by severe or systemic diseases (endocrine diseases, autoimmune diseases, infectious diseases, diabetes and other metabolic diseases) [6]; had brain surgery or metal implantation ; and [7] pregnant, breast-feeding, or having plans to conceive.

### Withdraw criteria

(1) The participant has an adverse event related to the research.

(2) At the participant’s own request.

### Randomization

Included participant will be randomly divided into the ARQE group and the CBTI group according to a 1:1 ratio. Block randomization is used with 4 block size to generate two different sets of random numbers lists. Random number tables are generated by researchers who have no contact with participants or evaluators. Each random number is sealed in a pre-prepared opaque envelope with the patient’s serial number on it. After the participant has been certified and signed a written informed consent, participant will open an envelope with patient serial number is opened and know the group of participant belongs to. Informed consent is available by email to corresponding authors. Because of the significant difference between massage treatment and CBTI, double-blinded method cannot be used. Thus, it is important to ensure the data analyst does not know the grouping and intervention method of the insomnia patients.

### Intervention

#### ARQE group

In this study, patients with insomnia received ARQE treatment 7 times a week for 8 weeks. A TCM doctor with more than 5 years of experience will intensively practice the patient once every Sunday. Each training lasts for 50 min, including breathing techniques and mild warm-up (10 min), abdomen-rubbing qigong exercise (30 min), and finally relax (10 min). Patients will be advised to exercise ARQE independently for 50 min before going to bed from Monday to Saturday.

**Fig. 2:**
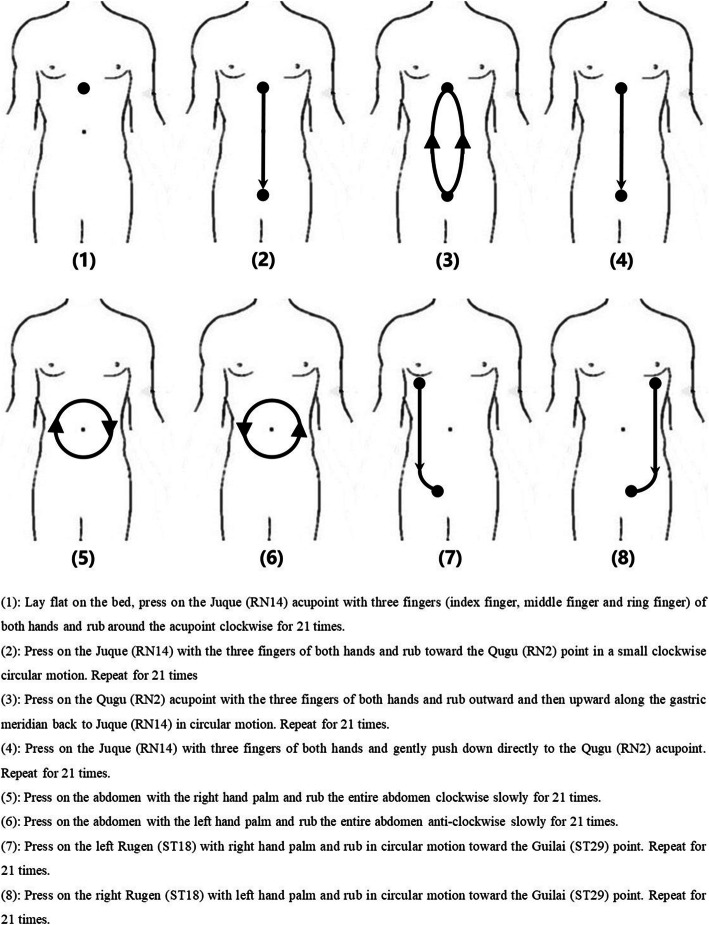
The abdomen-rubbing qigong exercise posture

In order to ensure the correct posture and movement speed, before the start of the experiment, the patient will receive 3 days of training provided by a TCM doctor with more than 5 years of experience to ensure the standard movements, strength, and speed of abdomen rubbing. The specific practice method of ARQE is shown in Fig. 2.

#### CBTI group

In this study, patients with insomnia will receive cognitive behavioral therapy for insomnia, including 8 weeks of cognitive behavioral therapy for insomnia with an experienced psychotherapist. The psychotherapist will provide weekly group cognitive behavioral therapy on Sundays. Patients were advised to conduct 50 min of self-behavioral cognitive learning every day from Monday to Saturday to ensure the same duration of intervention in the ARQE group. At the beginning of the experiment, we added the patient to the WeChat group organized by the psychotherapist and uploaded the lecture notes of the CBTI course to the WeChat group for the subjects to review. In addition, the therapist will regularly upload learning materials related to insomnia for the subjects to learn and regularly answer patients’ questions. Considering that Chinese people are generally unwilling to express their feelings in public, the psychotherapist will actively communicate with patients through social media (WeChat) once a week.

#### Follow up

After 8 weeks of intervention, all participants will enter an additional 4 weeks follow-up period. Participants will not receive any intervention during the follow-up period. Researchers will conduct a telephone follow-up or home visit in 4 weeks. The subjective feelings of the participants, PSQI, ISI, HAMD, and GSRS will be recorded.

#### Quality assurance

In order to ensure that patients in the ARQE group can practice the correct posture of ARQE by themselves at home, we will provide them with a video of the exercise method of ARQE recorded by a TCM doctor with 10 years of experience and a booklet with specific practice details. Participants will receive a “work practice record” and will be asked to fill it after each exercise.

### Outcome measures

To ensure the authenticity of the results, all questionnaires will be conducted by trained independent assessors. Regardless of whether the subjects complete the research project, we will collect the results of all participants. The study schedule (including enrollment, intervention, post-intervention, and follow-up period) is shown in Table 1.

#### Basic patient information

Basic information consists of the patient’s personal information and vital signs. Personal information includes age, gender, occupation, education level, and course of illness. Vital signs include body temperature, heart rate, respiratory rate, and blood pressure.

### Primary outcome

The primary outcome is the PSQI score. The PSQI will be assessed at week 0, week 4, week 8, and week 12. PSQI is composed of 19 sleep-related items and finally integrated into 7 dimensions: subjective sleep quality, time to fall asleep, sleep time, sleep efficiency, sleep disorder, hypnotic drugs, and daytime dysfunction. Each dimension is rated from 0 to 3 from mild and severe, with a total score of 21 points. A score more than 7 points to the presence of insomnia. The higher the total PSQI score is, the worse sleep quality [29].

### Secondary outcomes

Secondary outcomes include the Insomnia Severity Index (ISI), Gastrointestinal Symptom Scale (GSRS), Hamilton Depression Scale (HAMD), and rs-fMRI scan. ISI, GSRS, and HAMD will be assessed at week 0, week 4, week 8, and week 12. The rs-fMRI scan will be assessed at week 0 and week 8.

### Insomnia severity index (ISI)

ISI consists of 7 items to evaluate the degree of difficulty in falling asleep or maintaining asleep, that is, sleep satisfaction, daytime function, impaired attention due to sleep, the degree of pain of insomnia, and the degree of attention to insomnia, each of which is measured by five scales, with a total score ranged from 0 to 28 points, 0–7 points without insomnia, 8–14 points for mild insomnia, 15–21 points for moderate insomnia, and 22–28 points for severe insomnia [30].

### Gastrointestinal symptom scale (GSRS)

GSRS consists of 15 items, which are categorized into three groups, namely, gastric dysfunction, dyspepsia, and intestinal dysfunction. Each item is measure by seven scales, with a total score ranged from 0 to 105 points, used to evaluate the severity of gastrointestinal symptoms during the latest month. The higher the total score is, the more severe the symptoms are [31].

### Hamilton depression scale (HAMD)

HAMD is a scale used to assess depression. The version used in this study consists of 17 items, with a total score ranged from 0 to 53 points: 0–7 points without depression, 8–17 is mild depression, 17–24 is moderate depression, and > 24 is severe depression [32].

#### Neuroimaging

Fifteen participants of each group will receive rs-fMRI scan at weeks 0 and 8.

#### Rs-fMRI scan

The rs-fMRI data was collected by a 3.0 T Siemens MR scanner from Yueyang Hospital of Integrated Traditional Chinese and Western Medicine affiliated to Shanghai University of Traditional Chinese Medicine through a coil with a 32-channel head and neck coil. The sequence parameters of MRI are as follows: blood oxygen-dependent level fMRI (BLOD-fMRI), repetition time (TR) is 2000 ms, echo time (TE) is 30 mm, flip angle is 90°, and field of view (FOV) is 220 × 220 mm^2^; thickness/gap = 4.0/0.6 mm, and voxel size is 3 × 3 × 3. Participants should rest for half an hour before scanning and will be required to pay attention during the scanning process. All scanning operations are performed by relatively fixed radiation technicians and members of the research team during MRI operations. To ensure that the participants remain awake, the researchers will ask the subjects whether they are awake during and after the scan.

#### fMRI data preprocessing

Functional magnetic resonance data based on MATLAB 2013b (Math Works, Natick, MA, USA) platform pictures and picture parameter statistics software (http://www.fil.iom.ucl.ac.uk/spm, SPM12), static interest function Toolkit VBM8 (http://dbm.neuro.uni-jena.de/vbm8), and DPARSF V2.2 (http://rfinri.org/DPARSF ring state data processing assistant).

Data processing should be noted as follows: [1] removal of the data of the first ten points of time to ensure the stability of the signal, [2] slice timing correction: to eliminate the difference in fMRI signal interval scanning time, [3] head movement correction: to eliminate the artifacts generated by head motion and ensure the image quality, [4] spatial normalization to match the functional image with the structural image after head motion-correction to make it coincide with the standard brain, [5] reduction and elimination of noise generated in the previous step, [6] removal of the linear drift and exclusion the linear trend caused by the machine’s long time working temperature, and [7] filtering process to exclude high-frequency physiological noise and low-frequency drift.

### Composition of the coordinating center and trial steering committee

This project is directly supervised and managed by the research team of School of Acupuncture-Moxibustion and Tuina of Shanghai University of Traditional Chinese Medicine. The research team and special case inspectors from Yueyang Hospital of Integrated Traditional Chinese and Western Medicine affiliated to Shanghai University of Traditional Chinese Medicine set up a research steering group. The members of the Steering Group may conduct sampling checks on the completion of the case report form at any time.

### Composition of the data monitoring committee, its role, and reporting structure

The Data and Safety Monitoring Board (DSMB) will be composed of a physician, TCM doctor, medical statistician, ethicist, and radiologist and will oversee the study throughout the study period. The DSMB will supervise the safety data and clinical efficacy reports and monitor protocol compliance, data quality, and participant recruitment every month.

### Plans to promote participant retention and complete follow-up

To ensure retention of participants, they will be contacted by phone or WeChat 1 day before outcome assessment and will be reimbursed for round-trip travel. Participants have the right to withdraw at any stage of the study. When participants express a desire to withdraw from the study, the researchers will clarified any doubts and inform them of the potential benefits of being in the study. If a participant withdraws from the study, the researcher will try to contact the withdrawn participant to inquire about the reason for the withdrawal, whether there is an AE and outcome assessment.

### Safety assessments

If the participants experience any discomforts or unexpected situations during the clinical trial, they should inform the researchers immediately, regardless of the causes of these conditions. The researchers will do their best to prevent and treat any possible injuries caused by this study. Important AEs in this study would be qigong exercise-related AEs, such as headache, dizziness or vertigo, distension of head, tinnitus, stuffiness in the chest and worsening shortness of breath, heart-pounding or palpitations, muscular soreness or pain, profuse cold perspiration, irritability, neurasthenia, hallucination and paranoia, and psychological stress. Minor AEs will be treated immediately by the attending TCM doctor. In addition, all severe adverse events will be reported to the Regional Ethics Review Committee of Yueyang Hospital of Integrated Traditional Chinese and Western Medicine affiliated to Shanghai University of Traditional Chinese Medicine within 24 h. If adverse events are determined to be related to the study, the Regional Ethics Review Committee of Yueyang Hospital of Integrated Traditional Chinese and Western Medicine affiliated to Shanghai University of Traditional Chinese Medicine have the right to suspend this study. If an adverse event occurs during the clinical trial, medical experts will be recruited to evaluate and investigate the actual causes and medical and financial compensations will be made to the participant.

### Data management

Patients’ experimental data will be registered with electronic case report form separately by two researcher groups and verified by a third party. Any errors will be corrected and recorded immediately. All original data will be reserved for clinical data analysis and safety evaluation. All study-related data will be stored securely at the Shanghai University of Traditional Chinese Medicine.

### Statistical analysis

#### Primary outcome analysis

With previous set single blind rule, an independent statistician will analyze the clinical data by SPSS V.24.0 statistical software. Data analysis will be based on the intention-to-treat (ITT) principles. The results of ITT analysis will be compared for consistency. A *p* value < 0.05 will be considered statistically significant. Participants who fail to complete the study will be treated as having no change from baseline at all times in both groups. The Kolmogorov–Smirnov test will be used to test the normality. Appropriate transformations will be applied for non-normal distribution, The continuous variables with normal distribution will be described as mean and standard deviation, while continuous variables with non-normal distribution will be expressed by using median and interquartile interval (IQR), the categorical variable will be expressed in number and proportion.

To compare the baseline characteristics between two groups, Student’s *t* test or Mann–Whitney test will be used for continuous variables. Pearson’s *χ*-squared or Fisher’s exact will be used for categorical variables. To compare the primary and secondary outcomes between groups, continuous data will be tested by Student’s *t* test or non-parametric test. Pearson’s *χ*-squared or Fisher’s exact test will be applied for categorical data at baseline and after intervention. Repeated-measures ANOVA using the group-by-time interaction terms will be used to analyze the between-group differences. Multivariate analysis of variance will be used and the two-two comparisons among the changes in questionnaire scores with repeated measurement times for each group will be done by Bonferroni correction.

#### Secondary outcomes analysis

Secondary outcomes included clinical scale outcomes and neuroimage outcomes. Clinical scale outcomes will be undertaken by using a similar approach as the primary analysis.

The neuroimaging outcomes were statistically analyzed as follows: an independent *t* test will be used to compare the ALFF value and functional connection of the abdomen group, behavioral cognitive education group, and healthy control group. The two-sample *t* test will be used to compare the ALFF values between the two groups. Data of the two groups will be compared using paired *t* test before and after treatment. Select the largest peak coordinates of the different brain regions after ALFF analysis, save the clusters where the peak coordinates are located as a mask as the region of interest (ROI), and calculate the difference in functional connection between the ROI and the whole brain. The specific process is the seed area (ALFF difference brain area) is functionally connected with all voxels of the whole brain, which is the FC average image. After a correlation of values obtained from each voxel is subject to Fisher’s R-to-Z transformation, the data between groups will be subject to a two-sample *T* test and the data within the groups were subjected to paired *T* test and FWE correction. All the statistical analysis and processing will be completed independently by a research staff who has not participated in the treatment and scale evaluation throughout the entire process.

#### Interim analysis

An interim analysis will not be conducted.

#### Ethics and communication

Under the guidance of the Declaration of Helsinki’s general guidelines for medical research in humans and in accordance with local law, the study plan has been approved by the Regional Ethics Review Committee of Yueyang Hospital of Integrated Traditional Chinese and Western Medicine affiliated to Shanghai University of Traditional Chinese Medicine (Project NO. 2019-106) and registered on China Clinical Trial Registry (registration NO. ChiCTR1900028009). The standard protocol project: SPIRIT 2013 has also been observed [33]. Protocol modifications will be informed to Ethics Review Committees and the trial registry for their approval.

The results of this study will be published in a peer-reviewed journal or in the proceedings of academic conferences.

## Discussion

The purpose of this study is using the Clinical Scales and neuroimage scan to evaluate the clinical efficacy and brain function changes of the ARQE in the treatment of chronic insomnia. ARQE has sufficient theoretical basis and clinical practice experience in the treatment of insomnia. The classic Chinese traditional medicine “Huang Di Nei Jing” has the theory that “inharmonious stomach function leads to insomnia” [34, 35]. It is believed that gastrointestinal dysfunction may cause insomnia. Research has also found that gastrointestinal function and sleep affect each other. For example, patients with insomnia have abnormal digestive function, and patients with functional digestive tract diseases such as irritable bowel syndrome (IBS) and functional dyspepsia (FD) are also prone to sleep disorders [36]. Some research has also achieved good results in treating insomnia from the spleen and stomach theory. Wang’s research found that acupuncture at acupoints along the abdomen and stomach meridian of patients with chronic insomnia can relieve the symptoms of insomnia [37]. Zhang’s research found that acupuncture at local acupoints on the abdomen can relieve the clinical symptoms of patients [38].

ARQE is a treatment method mainly composed of 8 abdominal rubbing movements, which using gentle pressing to directly stimulate acupoints in the abdomen and stomach has the effect of enhancing digestive function and improving the efficacy of patients with insomnia symptoms. It has the characteristics of simple exercise method, gentle operation technique, flexible exercise time, and various postures. “Abdomen” is considered to be a specific acupuncture point for children’s massage in traditional Chinese medicine, and it is also often used in adult massage. It has the function of regulating the qi movement of the viscera and restoring the normal physiological functions of the viscera. There are a variety of internal organs in the abdomen, such as stomach, intestines, gallbladder, and pancreas. This gentle treatment can directly act on the abdomen and adjust the function of the gastrointestinal autonomic nerves by deforming and displacing the contents of the intestine, excites the intestinal parasympathetic nerves, promotes the contraction of gastrointestinal smooth muscles, strengthens peristalsis, and also promotes the secretion of gastric juice, bile, pancreatic juice, and small intestinal juice, and enhances the digestion of food by the stomach and the digestion and absorption of food by the small intestine. By improving the digestive function of patients, the symptoms of insomnia can be alleviated. In this trial, we asked patients with insomnia to perform supine ARQE before going to bed at night. This practice method helps patients relax before going to bed, reduces the time to fall asleep under gentle stimulation, and prepares the body and mind for sleep as soon as possible. ARQE, as a kind of qigong exercise, also has the characteristics of slow movement, coordination, and organic combination with breathing, which can regulate the negative emotions of patients with insomnia and change the activation of brain functions. In previous research, there is also a “brain-gut axis,” a neuro-endocrine network that connects the gastrointestinal tract and the brain through the nervous system. The central nervous system can regulate the function of the gastrointestinal tract by sending signal impulses down through the brain-gut axis [39]. Abnormal function of the gastrointestinal tract can also affect the related neural activities of the central or peripheral system including insomnia.

Chronic insomnia is a typical mind-body illness, which not only affects sleep function but also causes many other symptoms, such as mental health and digestive function. This study will evaluate the ARQE from multiple perspectives [40]. The most important thing in the treatment of insomnia patients with ARQE is to relieve the symptoms of insomnia. Therefore, in this study, PSQI and ISI scales which are most commonly used to evaluate sleep quality are selected to evaluate sleep quality and clinical effects of ARQE before and after intervention. Patients with insomnia often have some psychological and emotional abnormalities, which affect daytime function and aggravate the patient's symptoms. Therefore, HAMD will be used to assess the patients’ psychological depression. As a treatment method to treat insomnia from the spleen and stomach, we will use GSRS to reflect the gastrointestinal function of insomnia patients. We will observe changes in the gastrointestinal function of patients with insomnia before and after treatment to verify whether ARQE relieves the symptoms of insomnia by improving the gastrointestinal function and further verify the theory the theory of “inharmonious stomach function leads to insomnia”.

Neuroimage scan can reveal the changes in brain activation and functional connectivity in patients with insomnia treated by ARQE, providing clear, effective, and intuitive evidence. Previous fMRI studies have found abnormalities in multiple brain areas such as the cingulate gyrus, hippocampus, amygdala, insula, and prefrontal cortex in patients with insomnia [23]. Sleep disorders can lead to changes in the structure and function of the hippocampus [41, 42]. Patients with insomnia will experience bilateral hippocampal atrophy, involving the head, body, and tail of the hippocampus, and the size of the hippocampus is related to the length of the insomnia. In a recent study, the functional connectivity between the left hippocampus and the fusiform gyrus in patients with insomnia was weak [43]. After cognitive behavioral therapy, the functional connectivity between these altered left hippocampus and the fusiform gyrus was enhanced. These studies prove the important role of the hippocampus in the neuropathology of insomnia. Feng’s study found that after cupping intervention, the PSQI score of insomnia patients decreased the hippocampus volume increased and the brain function link between the hippocampus and the anterior cingulate cortex and the medial prefrontal cortex increased significantly [44]. Therefore, when studying ARQE, while observing the improvement of patients’ insomnia symptoms, we will also observe the correlation between the improvement of patients’ insomnia symptoms and the function of brain regions, especially the hippocampus and other important areas.

The results of this trial are expected to evaluate the efficacy of ARQE in the treatment of patients with insomnia through the degree of changes in brain activation and the related results of the clinical scale in order to confirm whether ARQE can effectively alleviate insomnia. We believe that the results of this study will provide a new solution for the treatment of insomnia for other researchers to learn from. This trial also has certain limitations. Due to the significant difference between “ARQE” and “CBTI,” blinding methods cannot be used for therapists and subjects, leading to a certain risk of bias in this experiment. Therefore, we set the same intensive training frequency to ensure the same frequency of contacting between the two groups of subjects and therapists, establish the same good doctor-patient relationship, and reduce the resulting differences. In order to ensure the authenticity of the experimental data and reduce the deviation due to the experimental process, we will strictly randomize the subjects and make the therapists, evaluators, and statisticians cannot contact each other. The neuroimaging research in this trial is only at the exploratory stage, and the sample size is limited. In future studies, the sample size can be increased to obtain more convincing evidence.

## Trial status

This protocol is version 1. Due to the time required for recruitment and preparation, the trial began in July 2020.The trial is currently in the early stages of recruiting participants. We expect the recruitment to be completed by July 2021.

## Data Availability

The protocol of the study is publicly available on the website of China Registered Clinical Trial Registration Center with no. ChiCTR1900021371. Any modification or adjustment of the experimental scheme will be submitted to the ethics committee for approval. The revised protocol will be submitted to the clinical registration center in time to apply for revision and complete the revision to inform all interested researchers. The datasets generated and/or analyzed during the current study are not publicly available due to China laws on privacy protection but are available from the corresponding author on reasonable request.

## Abbreviations

TCM: Traditional Chinese medicine
ARQE: Abdomen-rubbing qigong exercise
rs-fMRI: Resting state functional magnetic resonance imaging
fMRI: Functional magnetic resonance imaging
CBTI: Cognitive behavior therapy of insomnia
FC: Functional connectivity
ALFF: Amplitude of low-frequency amplitude
PSQI: Pittsburgh sleep quality Index
ISI: Insomnia severity index
GSRS: Gastrointestinal symptom scale
HAMD: Hamilton Depression Scale
ITT: Intention-to-treat
IQR: Interquartile interval
ROI: Region of interest
